# *Angiostrongylus cantonensis*: Lesions in Brain and Spinal Cord

**DOI:** 10.4269/ajtmh.2010.09-0658

**Published:** 2010-04

**Authors:** Zongli Diao, Erhu Jin, Chenghong Yin

**Affiliations:** Beijing Tropical Medicine Research Institute, and Department of Radiology, Beijing Friendship Hospital, Capital Medical University, Beijing, China

**Figure 1.**
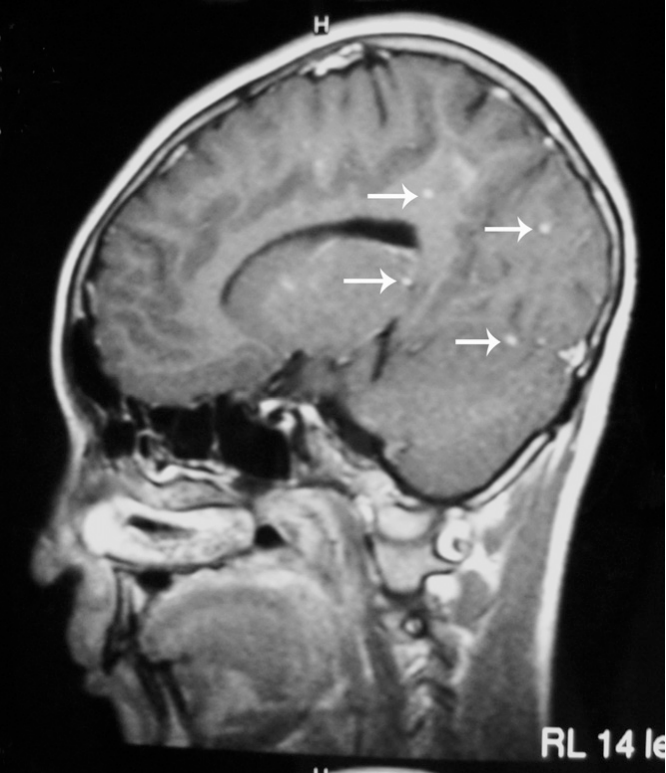
Magnetic resonance imaging of the brain of the patient, showing multiple lesions in sagittal T1-weighted images after administration of gadolinilum.

**Figure 2.**
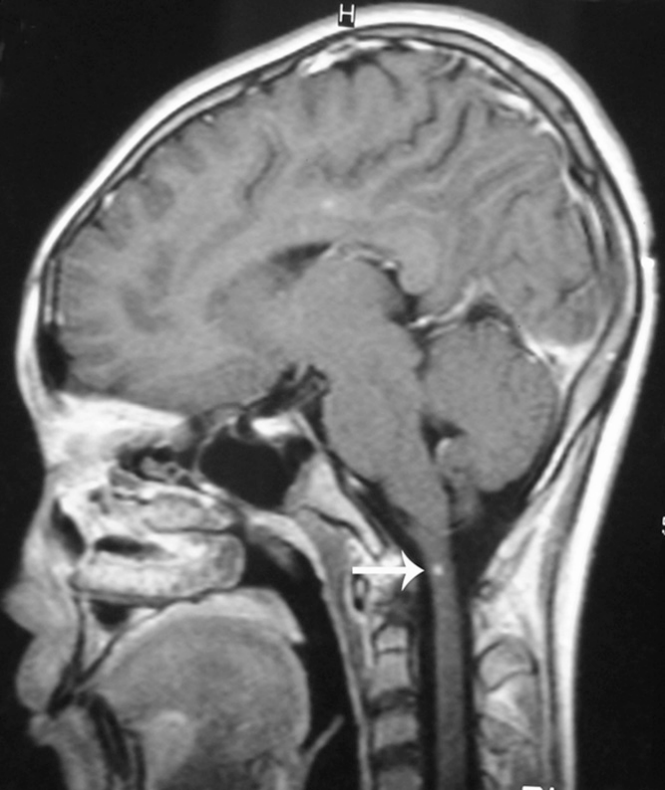
Spinal magnetic resonance imaging of the spine of the patient, showing a contrast-enhanced nodule in sagittal T1-weighted images after administration of gadolinilum.

A 13-year-old boy had a one-month history of left upper limb numbness and headache, and a 20-day history of intermittent fever. He had eaten an inadequately cooked *Pomacea canaliculata,* an intermediate host of *Angiostrongylus cantonensis*, 35 days earlier. Blood count analysis showed 7.52 × 10^9^ leukocytes/L with 18.3% eosinophils (0.5–5%). A lumbar puncture showed clear cerebrospinal fluid without erythrocytes (80 × 10^6^ cells/L), 45% lymphocytes and 54% eosinophils; the opening pressure was 150 mm of H_2_O. Results of tests for circulating antigens of *Angiostrongylus cantonensis* were positive. Magnetic resonance imaging of the brain showed an enhancement in sagittal T1-weighted images (Figure 1). Magnetic resonance imaging of the spine showed a contrast-enhanced nodule in sagittal T1-weighted images after administration of gadolinilum (Figure 2). Consequently, a diagnosis of angiostrongyliasis was made, and the patient was treated with albendazole and dexamethasone. Symptoms of headache and intermittent fever resolved within 20 days, but left upper limb numbness remained. This symptom disappeared with time. Brain and spinal cord lesions were completely resolved at a one-year follow-up.

